# Crystal structures and Hirshfeld surface analysis of 2-(adamantan-1-yl)-5-(4-fluoro­phen­yl)-1,3,4-oxa­diazole and 2-(adamantan-1-yl)-5-(4-chloro­phen­yl)-1,3,4-oxa­diazole

**DOI:** 10.1107/S2056989019004651

**Published:** 2019-04-12

**Authors:** Lamya H. Al-Wahaibi, Aisha Alsfouk, Ali A. El-Emam, Olivier Blacque

**Affiliations:** a Department of Chemistry, College of Sciences, Princess Nourah Bint Abdulrahman University, Riyadh 11671, Saudi Arabia; b Department of Pharmaceutical Sciences, College of Pharmacy, Princess Nourah Bint Abdulrahman University, Riyadh 11671, Saudi Arabia; c Department of Medicinal Chemistry, Faculty of Pharmacy, University of Mansoura, Mansoura 35516, Egypt; d Department of Chemistry, University of Zurich, Winterthurerstrasse 190, 8057 Zurich, Switzerland

**Keywords:** crystal structure, adamantyl derivatives, 1,3,4-oxa­diazo­les, C—H⋯N hydrogen bonds, C—H⋯F inter­actions, Hirshfeld surface analysis.

## Abstract

The crystal structures of the title adamantane-oxa­diazole hybrid compounds, C_18_H_19_FN_2_O (I) and C_18_H_19_ClN_2_O (II), are built up from an adamantane unit and a halogenophenyl ring, [*X* = F (I), Cl (II)], in position 5 on the central 1,3,4-oxa­diazole unit. The mol­ecular structures are very similar, only the relative orientation of the halogenophenyl ring in comparison with the central five-membered ring differs slightly. In the crystals of both compounds, mol­ecules are linked by pairs of C—H⋯N hydrogen bonds, forming inversion dimers with *R* ^2^_2_(12) ring motifs. In (I) the dimers are connected by C—H⋯F inter­actions, forming slabs lying parallel to the *bc* plane. In (II), the dimers are linked by C—H⋯π and offset π–π inter­actions [inter­planar distance = 3.4039 (9) Å], forming layers parallel to (10).

## Abstract 2

The title adamantane-oxa­diazole hybrid compounds are built up from an adamantane unit and a halogenophenyl ring, [*X* = F (I), Cl (II)], in position 5 on the central 1,3,4-oxa­diazole unit.

## Chemical context   

Considerable attention has been devoted to adamantane derivatives, which have long been known for their diverse biological properties (Liu *et al.*, 2011 ▸; Lamoureux & Artavia, 2010 ▸). In view of the pronounced lipophilicity of the adamantane cage, it has been observed that adamantyl-bearing compounds are characterized by high therapeutic indices (Wanka *et al.*, 2013 ▸). Sixty years ago, the first adamantane-based drug, amantadine, was discovered to be an efficient therapy for the treatment of Influenza A infection (Davies *et al.*, 1964 ▸; Togo *et al.*, 1968 ▸). As a result of intensive research based on adamantane derivatives, the adamantane nucleus was further recognized as the key pharmacophore in several biologically active compounds. Among the major biological activities displayed by adamantane derivatives, the anti-HIV (El-Emam *et al.*, 2004 ▸; Burstein *et al.*, 1999 ▸; Balzarini *et al.*, 2009 ▸), anti­bacterial (Protopopova *et al.*, 2005 ▸; El-Emam *et al.*, 2013 ▸; Kadi *et al.*, 2010 ▸; Al-Abdullah *et al.*, 2014 ▸; Al-Wahaibi *et al.*, 2017 ▸), anti­fungal (Omar *et al.*, 2010 ▸), anti­cancer (Sun *et al.*, 2002 ▸), anti-diabetic (Villhauer *et al.*, 2003 ▸; Augeri *et al.*, 2005 ▸) and anti­malarial (Dong *et al.*, 2010 ▸) activities are the most inter­esting. In addition, 1,3,4-oxa­diazole derivatives occupy a unique place in the field of medicinal chemistry as pharmacophores or auxophores possessing diverse pharmacological activities including anti­bacterial (Prakash *et al.*, 2010 ▸; Ogata *et al.*, 1971 ▸; Kadi *et al.*, 2007 ▸), anti­cancer (Zhang *et al.*, 2014 ▸), anti­viral (Wu *et al.*, 2015 ▸) and anti-inflammatory (Bansal *et al.*, 2014 ▸) activities. We report herein on the crystal structure determinations of the title adamantane-oxa­diazole hybrid mol­ecules 2-(adamantan-1-yl)-5-(4-fluoro­phen­yl)-1,3,4-oxa­diazole (I) and 2-(adamantan-1-yl)-5-(4-chloro­phen­yl)-1,3,4-oxa­diazole (II). The crystal structure of the 4-bromo­phenyl derivative has been reported previously (Alzoman *et al.*, 2014 ▸), and after examination of the deposited CIF and transformation of the space group, from *P*2_1_/*c* to *P*2_1_/*n*, it is found to be isotypic with compound (II).
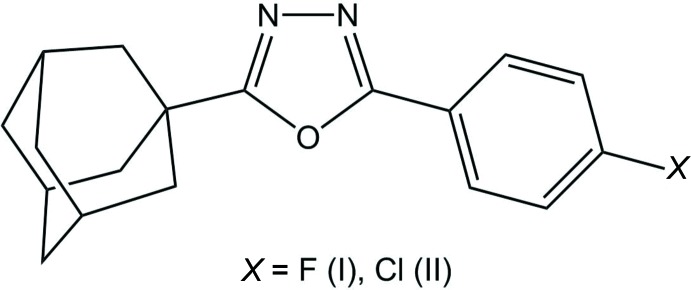


## Structural commentary   

**Figure 1:**
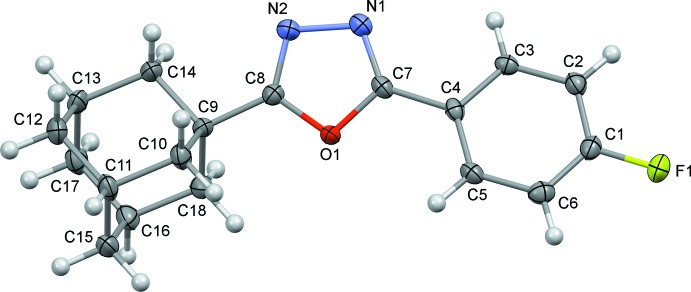
Mol­ecular structure of compound (I), with the atom labelling and displacement ellipsoids drawn at the 50% probability level.

Compounds (I) and (II), are built up from a central 1,3,4-oxa­diazole unit, an adamantane unit and a halogenophenyl group (Figs. 1 ▸ and 2 ▸, respectively). The C—N bonds in the oxa­diazole rings have double-bond character [C7=N1 = 1.279 (5) and 1.292 (3) Å, and C8=N2 = 1.288 (5) and 1.288 (3) Å in (I) and (II), respectively], while the N—N and C—O bonds exhibit single-bond character [N1—N2 = 1.408 (4) and 1.417 (3) Å, C7—O1 = 1.366 (4) and 1.360 (2) Å, and C8—O1 = 1.369 (4) and 1.359 (2) Å in (I) and (II), respectively]. These geometrical parameters are very similar to those observed for similar compounds; see §5. *Database survey*.

**Figure 3:**
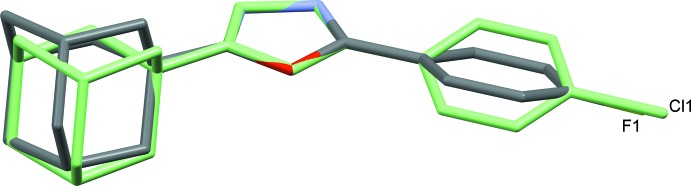
View of the structural overlay of compounds (I) and (II). Compound (I) is drawn according to element type, while compound (II) is drawn in pale green.

As seen in Fig. 3 ▸, the mol­ecular structures of compounds (I) and (II) are very similar. The largest difference is highlighted by the structural overlay plot, and comes from the relative orientation of the halogenophenyl group with respect to the oxa­diazole ring. In compound (II), the rings are almost coplanar with their mean planes being inclined to each other by 9.5 (1)°, while in compound (I) the equivalent dihedral angle is 20.8 (2)°.

## Supra­molecular features   

**Figure 4:**
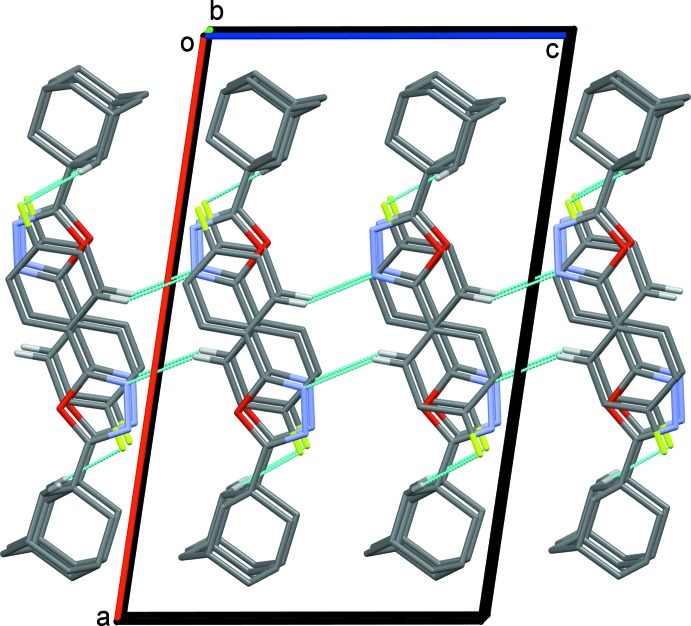
A view along the *b* axis of the crystal packing of compound (I). The hydrogen-bonding inter­actions (see Table 1 ▸) are shown as dashed lines. For clarity, only hydrogen atoms H3 and H18*A* have been included.

**Figure 5:**
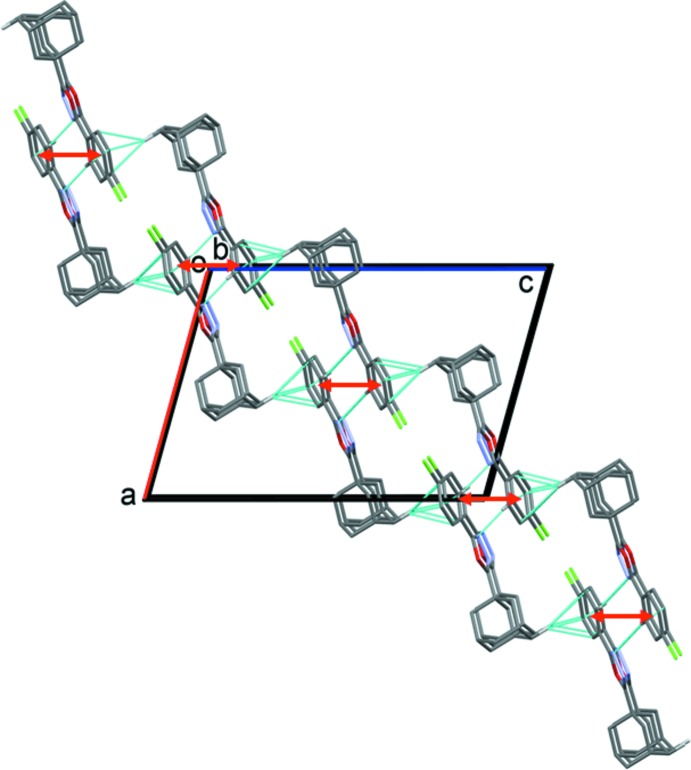
A view along the *b* axis of the crystal packing of compound (II), showing the C—H⋯N hydrogen bonds and the C—H⋯π inter­actions (see Table 2 ▸) as dashed lines. The offset π–π inter­actions are indicated by double-headed red arrows. For clarity, only hydrogen atoms H3 and H12*A* have been included.

In the crystals of both compounds, mol­ecules are linked by pairs of C—H⋯N hydrogen bonds, forming inversion dimers with 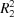
(12) ring motifs (Tables 1 ▸ and 2 ▸, respectively). In the crystal of (I), the dimers are connected by C—H⋯F inter­actions, forming slabs lying parallel to the *bc* plane (Fig. 4 ▸ and Table 1 ▸). In the crystal of (II), the dimers are linked by C—H⋯π and offset π–π inter­actions, forming layers lying parallel to the (10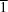) plane; see Fig. 5 ▸ and Table 2 ▸. The offset π–π inter­actions involve inversion-related 4-chloro­phenyl rings (C1–C6) with an inter­centroid distance of 3.687 (1) Å, an inter­planar distance of 3.404 (1) Å, and an offset of 1.418 Å. In Fig. 5 ▸ these inter­actions are represented by double-headed red arrows.

## Hirshfeld surface analysis   

**Figure 6:**
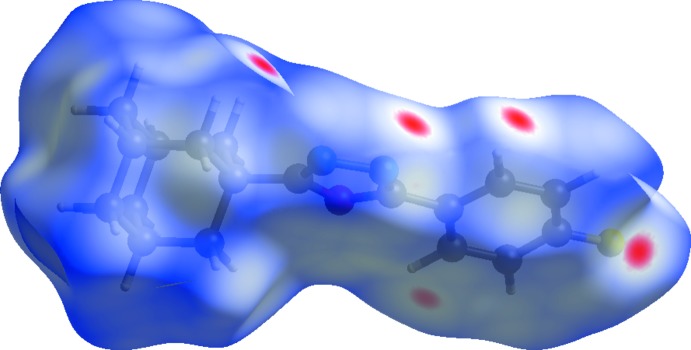
A view of the Hirshfeld surface mapped over *d*_norm_ for compound (I) over the range −0.138 to 1.364 arbitrary units.

**Figure 7:**
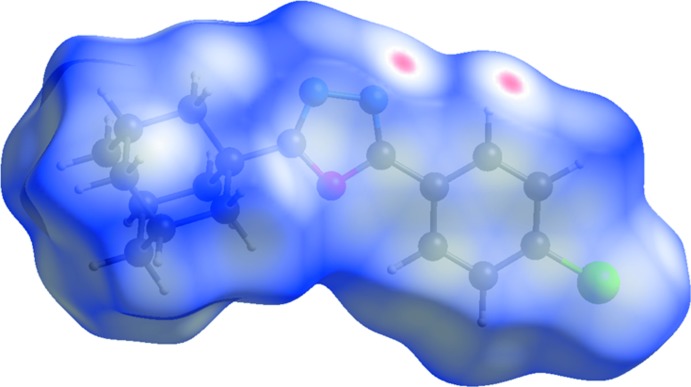
A view of the Hirshfeld surface mapped over *d*_norm_ for compound (II) over the range −0.203 to 1.273 arbitrary units.

**Figure 8:**
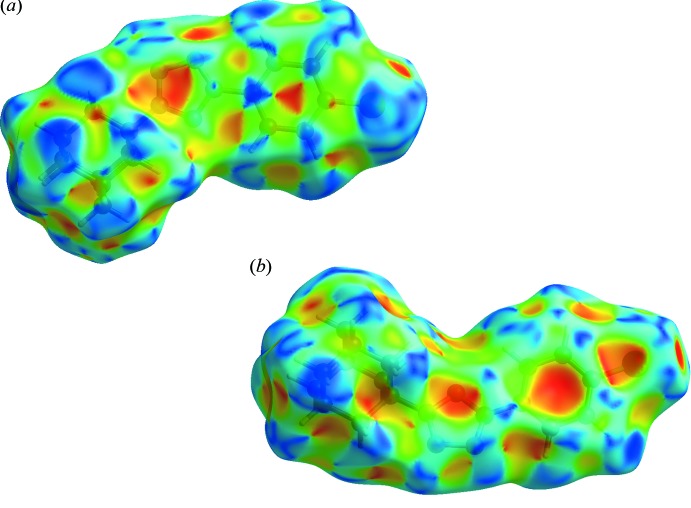
Two views, (*a*) and (*b*), of the Hirshfeld surface mapped over the shape-index property for compound (II).

The Hirshfeld surfaces for (I) and (II) mapped over *d*_norm_ were calculated using *CrystalExplorer 17* (Turner *et al.*, 2017 ▸) with the default setting of arbitrary units range. The characteristic bright-red spots near atoms H3, H18*A*, N1 and F1 (Fig. 6 ▸) confirm the previously mentioned C3—H3⋯N1^i^ and C18—H18*A*⋯F1^ii^ [symmetry codes: (i) −*x* + 1, −*y* + 1, −*z* + 1; (ii) −*x* + 1, *y* − 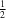, −*z* + 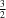] inter­atomic contacts in the crystal packing of (I). As expected, the same bright-red spots are observed near atoms H3 and N1 on the Hirshfeld surface of (II); see Fig. 7 ▸. The Hirshfeld surface mapped over the shape-index property elegantly illustrates the π–π stacking and the C—H⋯π inter­actions observed in the crystal packing of (II). Two views are presented in Fig. 8 ▸. The π–π stacking between inversion-related 4-chloro­phenyl rings (C1–C6) is indicated by the appearance of small blue regions surrounding a bright-red triangle within the six-membered ring (Fig. 8 ▸ *a*), while the C12—H12*A*⋯π(C1–C6)^iii^ inter­action [symmetry code: (iii) *x* + 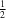, −*y* + 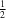, *z* + 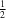] appears as a large red region within the ring (Fig. 8 ▸ *b*).

## Database survey   

A search of the Cambridge Structural Database (CSD, version 5.40, February 2019; Groom *et al.*, 2016 ▸) for the substructure 2-(adamantan-1-yl)-1,3,4-oxa­diazole gave five hits. The crystal structures of three very similar compounds were reported in the last decade, namely 2-(adamantan-1-yl)-5-(4-nitro­phen­yl)-1,3,4-oxa­diazole (CSD refcode LAPVOP; El-Emam *et al.*, 2012 ▸), which has an NO_2_ group on the phenyl ring (in the *para* position to the oxa­diazole ring), 2-(adamantan-1-yl)-5-(4-bromo­phen­yl)-1,3,4-oxa­diazole (SOSXIJ; Alzoman *et al.* 2014 ▸), which has a Br atom on the phenyl ring (same *para* position) and 2-(adamantan-1-yl)-5-(3-fluoro­phen­yl)-1,3,4-oxa­diazole (SIKKAA; Khan *et al.*, 2012 ▸), with a 3-fluoro­phenyl substituent at position 5 on the oxa­diazole ring. Two more recently reported structures are (5-(adamantan-1-yl)-1,3,4-oxa­diazole-2-thiol­ato)tri­phenyl­phosphinegold(I) (AZECAL; Garcia *et al.*, 2016 ▸) and 2-(adamantan-1-yl)-5-[2-(2-methyl­phen­yl)-1,3-thia­zol-4-yl]-1,3,4-oxa­diazole (XARGEE­01; Khan *et al.*, 2016 ▸).

The reduced cell of SOSXIJ indicates that it is isotypic with compound (II). Compound LAPVOP resides on a mirror plane, while compound SIKKAA crystallizes with two independent mol­ecules in the asymmetric unit. The geometrical parameters of the oxa­diazole rings are similar to those reported above for the title compounds. The 4-substituted phenyl rings are inclined to the oxa­diazole ring by 0.0° in LAPVOP (as it lies in a mirror plane), 3.01 and 3.31° in the two independent mol­ecules of SIKKAA and 10.44° in SOSXIJ. In the title compounds the corresponding dihedral angle is 20.8 (2)° for compound (I) and 9.5 (1)° for compound (II).

## Synthesis and crystallization   

Compounds (I) and (II) were synthesized *via* condensation of adamantane-1-carb­oxy­lic acid with 4-fluoro­benzohydrazide, or 4-chloro­benzohydrazide in the presence of phospho­rus oxychloride, as described previously (Kadi *et al.*, 2007 ▸). Colourless plate-like crystals of compound (I) and colourless needle-like crystals of compound (II) were obtained by slow evaporation of CHCl_3_:EtOH (1:1 *v*:*v*) solutions at room temperature.

## Refinement   

Crystal data, data collection and structure refinement details are summarized in Table 3 ▸. All H atoms were placed in calculated positions and treated as riding atoms: C—H = 0.95–1.00 Å with *U*_iso_ = 1.2*U*_eq_(C).

## Supplementary Material

Crystal structure: contains datablock(s) I, II, Global. DOI: 10.1107/S2056989019004651/su5492sup1.cif

Structure factors: contains datablock(s) I. DOI: 10.1107/S2056989019004651/su5492Isup4.hkl

Structure factors: contains datablock(s) II. DOI: 10.1107/S2056989019004651/su5492IIsup5.hkl

Supporting information file. DOI: 10.1107/S2056989019004651/su5492Isup4.cml

Supporting information file. DOI: 10.1107/S2056989019004651/su5492IIsup5.cml

CCDC references: 1908204, 1908203

Additional supporting information:  crystallographic information; 3D view; checkCIF report

## Figures and Tables

**Figure 2:**
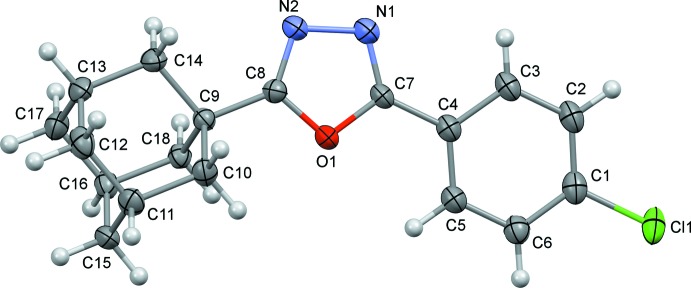
Mol­ecular structure of compound (II), with the atom labelling and displacement ellipsoids drawn at the 50% probability level.

**Table 1:** Hydrogen-bond geometry (Å, °) for (I) *Cg*1 is the centroid of the C1–C6 ring.

*D*—H⋯*A*	*D*—H	H⋯*A*	*D*⋯*A*	*D*—H⋯*A*
C3—H3⋯N1^i^	0.95	2.56	3.383 (5)	146
C18—H18*A*⋯F1^ii^	0.99	2.47	3.415 (5)	159

Symmetry codes: (i) 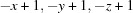; (ii) 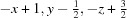.

**Table 2:** Hydrogen-bond geometry (Å, °) for (II) *Cg*1 is the centroid of the C1–C6 ring.

*D*—H⋯*A*	*D*—H	H⋯*A*	*D*⋯*A*	*D*—H⋯*A*
C3—H3⋯N1^i^	0.95	2.61	3.386 (3)	139
C12—H12*A*⋯*Cg*1^ii^	0.99	2.73	3.680 (3)	160

Symmetry codes: (i) 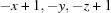; (ii) 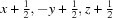.

**Table 3:** Experimental details

	(I)	(II)
Crystal data		
Chemical formula	C_18_H_19_FN_2_O	C_18_H_19_ClN_2_O
*M*_r_	298.35	314.80
Crystal system, space group	Monoclinic, *P*2_1_/*c*	Monoclinic, *P*2_1_/*n*
Temperature (K)	160	160
*a*, *b*, *c* (Å)	18.2525 (4), 7.07855 (16), 11.2207 (2)	13.08241 (19), 6.49259 (9), 18.5129 (3)
β (°)	98.556 (2)	105.5609 (16)
*V* (Å^3^)	1433.59 (6)	1514.83 (4)
*Z*	4	4
Radiation type	Cu *K*α	Cu *K*α
μ (mm^−1^)	0.78	2.25
Crystal size (mm)	0.18 × 0.15 × 0.02	0.33 × 0.12 × 0.08

Data collection		
Diffractometer	XtaLAB Synergy, Dualflex, Pilatus 200K	XtaLAB Synergy, Dualflex, Pilatus 200K
Absorption correction	Analytical (*CrysAlis PRO*; Rigaku OD, 2019 ▸)	Analytical (*CrysAlis PRO*; Rigaku OD, 2019 ▸)
*T*_min_, *T*_max_	0.921, 0.990	0.642, 0.870
No. of measured, independent and observed [*I* > 2σ(*I*)] reflections	13058, 2903, 2578	14318, 3217, 3052
*R*_int_	0.030	0.023
(sin θ/λ)_max_ (Å^−1^)	0.625	0.636

Refinement		
*R*[*F* ^2^ > 2σ(*F* ^2^)], *wR*(*F* ^2^), *S*	0.080, 0.233, 1.23	0.061, 0.167, 1.08
No. of reflections	2903	3217
No. of parameters	199	199
H-atom treatment	H-atom parameters constrained	H-atom parameters constrained
Δρ_max_, Δρ_min_ (e Å^−3^)	0.47, −0.38	0.81, −0.26

Computer programs: *CrysAlis PRO* (Rigaku OD, 2019 ▸), *SHELXT* (Sheldrick, 2015*a* ▸), *SHELXL* (Sheldrick, 2015*b* ▸), *Mercury* (Macrae *et al.*, 2008 ▸) and *OLEX2* (Dolomanov *et al.*, 2009 ▸).

## supplementary crystallographic information

### 2-(Adamantan-1-yl)-5-(4-fluorophenyl)-1,3,4-oxadiazole (I) Crystal data

**Table:**

C_18_H_19_FN_2_O	*F*(000) = 632
*M**_r_* = 298.35	*D*_x_ = 1.382 Mg m^−^^3^
Monoclinic, *P*2_1_/*c*	Cu *K*α radiation, λ = 1.54184 Å
*a* = 18.2525 (4) Å	Cell parameters from 7588 reflections
*b* = 7.07855 (16) Å	θ = 4.9–78.5°
*c* = 11.2207 (2) Å	µ = 0.78 mm^−^^1^
β = 98.556 (2)°	*T* = 160 K
*V* = 1433.59 (6) Å^3^	Plate, colourless
*Z* = 4	0.18 × 0.15 × 0.02 mm

### 2-(Adamantan-1-yl)-5-(4-fluorophenyl)-1,3,4-oxadiazole (I) Data collection

**Table:**

XtaLAB Synergy, Dualflex, Pilatus 200K diffractometer	2903 independent reflections
Radiation source: micro-focus sealed X-ray tube, PhotonJet (Cu) X-ray Source	2578 reflections with *I* > 2σ(*I*)
Mirror monochromator	*R*_int_ = 0.030
Detector resolution: 5.8140 pixels mm^-1^	θ_max_ = 74.5°, θ_min_ = 4.9°
ω scans	*h* = −22→22
Absorption correction: analytical (CrysAlisPro; Rigaku OD, 2019)	*k* = −8→8
*T*_min_ = 0.921, *T*_max_ = 0.990	*l* = −11→14
13058 measured reflections	

### 2-(Adamantan-1-yl)-5-(4-fluorophenyl)-1,3,4-oxadiazole (I) Refinement

**Table:**

Refinement on *F*^2^	Primary atom site location: dual
Least-squares matrix: full	Hydrogen site location: inferred from neighbouring sites
*R*[*F*^2^ > 2σ(*F*^2^)] = 0.080	H-atom parameters constrained
*wR*(*F*^2^) = 0.233	*w* = 1/[σ^2^(*F*_o_^2^) + (0.0572*P*)^2^ + 6.0123*P*] where *P* = (*F*_o_^2^ + 2*F*_c_^2^)/3
*S* = 1.23	(Δ/σ)_max_ < 0.001
2903 reflections	Δρ_max_ = 0.47 e Å^−^^3^
199 parameters	Δρ_min_ = −0.38 e Å^−^^3^
0 restraints	

### 2-(Adamantan-1-yl)-5-(4-fluorophenyl)-1,3,4-oxadiazole (I) Special details

**Table:**

Geometry. All esds (except the esd in the dihedral angle between two l.s. planes) are estimated using the full covariance matrix. The cell esds are taken into account individually in the estimation of esds in distances, angles and torsion angles; correlations between esds in cell parameters are only used when they are defined by crystal symmetry. An approximate (isotropic) treatment of cell esds is used for estimating esds involving l.s. planes.

### 2-(Adamantan-1-yl)-5-(4-fluorophenyl)-1,3,4-oxadiazole (I) Fractional atomic coordinates and isotropic or equivalent isotropic displacement parameters (Å^2^)

**Table:**

	*x*	*y*	*z*	*U*_iso_*/*U*_eq_	
C1	0.6397 (2)	0.5834 (6)	0.8751 (3)	0.0220 (8)	
C2	0.6287 (2)	0.5485 (6)	0.7528 (3)	0.0218 (8)	
H2	0.669009	0.519350	0.711237	0.026*	
C3	0.5568 (2)	0.5574 (5)	0.6928 (3)	0.0189 (7)	
H3	0.547397	0.536452	0.608242	0.023*	
C4	0.4979 (2)	0.5973 (5)	0.7558 (3)	0.0178 (7)	
C5	0.5114 (2)	0.6345 (5)	0.8784 (3)	0.0201 (8)	
H5	0.471482	0.664740	0.920495	0.024*	
C6	0.5836 (2)	0.6276 (6)	0.9397 (3)	0.0234 (8)	
H6	0.593767	0.652639	1.023700	0.028*	
C7	0.42268 (19)	0.5993 (5)	0.6869 (3)	0.0188 (7)	
C8	0.3054 (2)	0.5755 (5)	0.6523 (3)	0.0194 (8)	
C9	0.22741 (19)	0.5532 (5)	0.6776 (3)	0.0188 (8)	
C10	0.2034 (2)	0.7286 (6)	0.7450 (4)	0.0229 (8)	
H10A	0.235907	0.742685	0.823383	0.027*	
H10B	0.208148	0.843843	0.696746	0.027*	
C11	0.1226 (2)	0.7041 (6)	0.7656 (4)	0.0249 (9)	
H11	0.107002	0.816945	0.809285	0.030*	
C12	0.0729 (2)	0.6855 (7)	0.6436 (4)	0.0280 (9)	
H12A	0.020522	0.672769	0.656054	0.034*	
H12B	0.077248	0.800506	0.594894	0.034*	
C13	0.0958 (2)	0.5124 (6)	0.5766 (3)	0.0261 (9)	
H13	0.063156	0.501154	0.496999	0.031*	
C14	0.1769 (2)	0.5336 (6)	0.5561 (3)	0.0241 (8)	
H14A	0.191844	0.421626	0.512660	0.029*	
H14B	0.181982	0.646678	0.506007	0.029*	
C15	0.1155 (2)	0.5267 (7)	0.8408 (4)	0.0282 (9)	
H15A	0.147857	0.538200	0.919522	0.034*	
H15B	0.063748	0.512872	0.855987	0.034*	
C16	0.1378 (2)	0.3532 (6)	0.7743 (4)	0.0264 (9)	
H16	0.132697	0.237763	0.823688	0.032*	
C17	0.0881 (2)	0.3342 (6)	0.6516 (4)	0.0282 (9)	
H17A	0.102861	0.221647	0.608557	0.034*	
H17B	0.035805	0.318109	0.663834	0.034*	
C18	0.2193 (2)	0.3748 (6)	0.7544 (4)	0.0242 (8)	
H18A	0.234847	0.261751	0.712615	0.029*	
H18B	0.251650	0.386012	0.833140	0.029*	
F1	0.71024 (12)	0.5714 (4)	0.9363 (2)	0.0321 (6)	
N1	0.40489 (17)	0.6139 (5)	0.5727 (3)	0.0237 (7)	
N2	0.32721 (18)	0.5975 (5)	0.5493 (3)	0.0245 (7)	
O1	0.36296 (13)	0.5751 (4)	0.7457 (2)	0.0192 (6)	

### 2-(Adamantan-1-yl)-5-(4-fluorophenyl)-1,3,4-oxadiazole (I) Atomic displacement parameters (Å^2^)

**Table:**

	*U*^11^	*U*^22^	*U*^33^	*U*^12^	*U*^13^	*U*^23^
C1	0.0185 (18)	0.0220 (19)	0.0239 (19)	−0.0016 (15)	−0.0015 (14)	0.0036 (15)
C2	0.0179 (17)	0.0239 (19)	0.0245 (19)	−0.0014 (15)	0.0059 (14)	0.0014 (15)
C3	0.0218 (18)	0.0180 (17)	0.0177 (17)	0.0000 (14)	0.0055 (14)	−0.0012 (13)
C4	0.0152 (16)	0.0155 (16)	0.0232 (17)	−0.0024 (13)	0.0048 (13)	0.0008 (14)
C5	0.0199 (17)	0.0201 (18)	0.0217 (18)	0.0007 (14)	0.0083 (14)	0.0003 (14)
C6	0.028 (2)	0.0220 (19)	0.0204 (18)	−0.0023 (16)	0.0029 (15)	−0.0004 (15)
C7	0.0170 (17)	0.0187 (17)	0.0220 (18)	−0.0004 (14)	0.0074 (14)	0.0000 (14)
C8	0.0182 (17)	0.0217 (19)	0.0178 (17)	0.0005 (14)	0.0014 (13)	−0.0002 (14)
C9	0.0163 (17)	0.0260 (19)	0.0141 (16)	0.0021 (14)	0.0015 (13)	0.0001 (14)
C10	0.0163 (18)	0.027 (2)	0.0257 (19)	−0.0027 (15)	0.0034 (14)	−0.0046 (16)
C11	0.0153 (17)	0.034 (2)	0.027 (2)	0.0003 (16)	0.0061 (14)	−0.0057 (17)
C12	0.0159 (18)	0.039 (2)	0.029 (2)	0.0032 (17)	0.0029 (15)	0.0023 (18)
C13	0.0170 (18)	0.041 (2)	0.0191 (18)	−0.0018 (16)	−0.0021 (14)	−0.0039 (17)
C14	0.0179 (18)	0.038 (2)	0.0161 (17)	−0.0002 (16)	0.0018 (14)	−0.0001 (16)
C15	0.0197 (18)	0.045 (3)	0.0201 (19)	−0.0023 (17)	0.0047 (14)	−0.0018 (18)
C16	0.0197 (18)	0.031 (2)	0.030 (2)	−0.0026 (16)	0.0057 (15)	0.0047 (17)
C17	0.0205 (19)	0.034 (2)	0.030 (2)	−0.0060 (17)	0.0046 (16)	−0.0061 (18)
C18	0.0211 (18)	0.029 (2)	0.0233 (19)	0.0043 (16)	0.0051 (15)	0.0048 (16)
F1	0.0198 (11)	0.0465 (16)	0.0278 (12)	−0.0006 (10)	−0.0032 (9)	0.0031 (11)
N1	0.0194 (15)	0.0318 (18)	0.0207 (16)	0.0022 (13)	0.0061 (12)	0.0008 (14)
N2	0.0202 (16)	0.0366 (19)	0.0171 (15)	0.0020 (14)	0.0044 (12)	0.0013 (14)
O1	0.0148 (12)	0.0288 (14)	0.0146 (12)	−0.0007 (10)	0.0036 (9)	−0.0009 (10)

### 2-(Adamantan-1-yl)-5-(4-fluorophenyl)-1,3,4-oxadiazole (I) Geometric parameters (Å, º)

**Table:**

C1—C2	1.380 (5)	C11—H11	1.0000
C1—C6	1.376 (5)	C11—C12	1.532 (6)
C1—F1	1.369 (4)	C11—C15	1.529 (6)
C2—H2	0.9500	C12—H12A	0.9900
C2—C3	1.384 (5)	C12—H12B	0.9900
C3—H3	0.9500	C12—C13	1.528 (6)
C3—C4	1.400 (5)	C13—H13	1.0000
C4—C5	1.386 (5)	C13—C14	1.539 (5)
C4—C7	1.472 (5)	C13—C17	1.535 (6)
C5—H5	0.9500	C14—H14A	0.9900
C5—C6	1.394 (5)	C14—H14B	0.9900
C6—H6	0.9500	C15—H15A	0.9900
C7—N1	1.279 (5)	C15—H15B	0.9900
C7—O1	1.366 (4)	C15—C16	1.523 (6)
C8—C9	1.500 (5)	C16—H16	1.0000
C8—N2	1.288 (5)	C16—C17	1.538 (6)
C8—O1	1.369 (4)	C16—C18	1.544 (5)
C9—C10	1.550 (5)	C17—H17A	0.9900
C9—C14	1.534 (5)	C17—H17B	0.9900
C9—C18	1.548 (5)	C18—H18A	0.9900
C10—H10A	0.9900	C18—H18B	0.9900
C10—H10B	0.9900	N1—N2	1.408 (4)
C10—C11	1.537 (5)		
			
C6—C1—C2	123.8 (4)	H12A—C12—H12B	108.2
F1—C1—C2	118.3 (3)	C13—C12—C11	109.8 (3)
F1—C1—C6	117.9 (3)	C13—C12—H12A	109.7
C1—C2—H2	121.2	C13—C12—H12B	109.7
C1—C2—C3	117.5 (3)	C12—C13—H13	109.5
C3—C2—H2	121.2	C12—C13—C14	109.6 (3)
C2—C3—H3	119.8	C12—C13—C17	109.6 (3)
C2—C3—C4	120.5 (3)	C14—C13—H13	109.5
C4—C3—H3	119.8	C17—C13—H13	109.5
C3—C4—C7	117.6 (3)	C17—C13—C14	109.3 (3)
C5—C4—C3	120.2 (3)	C9—C14—C13	109.9 (3)
C5—C4—C7	122.2 (3)	C9—C14—H14A	109.7
C4—C5—H5	120.1	C9—C14—H14B	109.7
C4—C5—C6	119.9 (3)	C13—C14—H14A	109.7
C6—C5—H5	120.1	C13—C14—H14B	109.7
C1—C6—C5	118.1 (3)	H14A—C14—H14B	108.2
C1—C6—H6	121.0	C11—C15—H15A	109.7
C5—C6—H6	121.0	C11—C15—H15B	109.7
N1—C7—C4	127.2 (3)	H15A—C15—H15B	108.2
N1—C7—O1	113.1 (3)	C16—C15—C11	110.0 (3)
O1—C7—C4	119.7 (3)	C16—C15—H15A	109.7
N2—C8—C9	127.7 (3)	C16—C15—H15B	109.7
N2—C8—O1	112.5 (3)	C15—C16—H16	109.4
O1—C8—C9	119.8 (3)	C15—C16—C17	110.1 (3)
C8—C9—C10	110.7 (3)	C15—C16—C18	108.9 (3)
C8—C9—C14	107.7 (3)	C17—C16—H16	109.4
C8—C9—C18	111.2 (3)	C17—C16—C18	109.5 (3)
C14—C9—C10	109.3 (3)	C18—C16—H16	109.4
C14—C9—C18	109.1 (3)	C13—C17—C16	109.2 (3)
C18—C9—C10	108.9 (3)	C13—C17—H17A	109.8
C9—C10—H10A	109.8	C13—C17—H17B	109.8
C9—C10—H10B	109.8	C16—C17—H17A	109.8
H10A—C10—H10B	108.3	C16—C17—H17B	109.8
C11—C10—C9	109.3 (3)	H17A—C17—H17B	108.3
C11—C10—H10A	109.8	C9—C18—H18A	109.8
C11—C10—H10B	109.8	C9—C18—H18B	109.8
C10—C11—H11	109.4	C16—C18—C9	109.6 (3)
C12—C11—C10	109.2 (3)	C16—C18—H18A	109.8
C12—C11—H11	109.4	C16—C18—H18B	109.8
C15—C11—C10	109.7 (3)	H18A—C18—H18B	108.2
C15—C11—H11	109.4	C7—N1—N2	106.2 (3)
C15—C11—C12	109.6 (3)	C8—N2—N1	106.3 (3)
C11—C12—H12A	109.7	C7—O1—C8	102.0 (3)
C11—C12—H12B	109.7		
			
C1—C2—C3—C4	−1.1 (6)	C11—C15—C16—C17	−59.2 (4)
C2—C1—C6—C5	1.1 (6)	C11—C15—C16—C18	60.8 (4)
C2—C3—C4—C5	2.2 (6)	C12—C11—C15—C16	59.0 (4)
C2—C3—C4—C7	−178.3 (3)	C12—C13—C14—C9	59.4 (4)
C3—C4—C5—C6	−1.6 (6)	C12—C13—C17—C16	−59.5 (4)
C3—C4—C7—N1	−18.2 (6)	C14—C9—C10—C11	59.8 (4)
C3—C4—C7—O1	158.6 (3)	C14—C9—C18—C16	−59.3 (4)
C4—C5—C6—C1	0.0 (6)	C14—C13—C17—C16	60.6 (4)
C4—C7—N1—N2	176.5 (4)	C15—C11—C12—C13	−59.5 (4)
C4—C7—O1—C8	−176.9 (3)	C15—C16—C17—C13	59.3 (4)
C5—C4—C7—N1	161.3 (4)	C15—C16—C18—C9	−60.5 (4)
C5—C4—C7—O1	−22.0 (5)	C17—C13—C14—C9	−60.7 (4)
C6—C1—C2—C3	−0.5 (6)	C17—C16—C18—C9	59.9 (4)
C7—C4—C5—C6	178.9 (4)	C18—C9—C10—C11	−59.3 (4)
C7—N1—N2—C8	0.4 (4)	C18—C9—C14—C13	59.8 (4)
C8—C9—C10—C11	178.1 (3)	C18—C16—C17—C13	−60.5 (4)
C8—C9—C14—C13	−179.4 (3)	F1—C1—C2—C3	178.7 (3)
C8—C9—C18—C16	−177.9 (3)	F1—C1—C6—C5	−178.2 (3)
C9—C8—N2—N1	179.3 (4)	N1—C7—O1—C8	0.3 (4)
C9—C8—O1—C7	−179.6 (3)	N2—C8—C9—C10	−111.7 (4)
C9—C10—C11—C12	−60.3 (4)	N2—C8—C9—C14	7.7 (6)
C9—C10—C11—C15	59.8 (4)	N2—C8—C9—C18	127.1 (4)
C10—C9—C14—C13	−59.2 (4)	N2—C8—O1—C7	−0.1 (4)
C10—C9—C18—C16	59.8 (4)	O1—C7—N1—N2	−0.4 (4)
C10—C11—C12—C13	60.7 (4)	O1—C8—C9—C10	67.8 (4)
C10—C11—C15—C16	−60.9 (4)	O1—C8—C9—C14	−172.9 (3)
C11—C12—C13—C14	−60.0 (4)	O1—C8—C9—C18	−53.4 (4)
C11—C12—C13—C17	60.0 (4)	O1—C8—N2—N1	−0.2 (4)

### 2-(Adamantan-1-yl)-5-(4-fluorophenyl)-1,3,4-oxadiazole (I) Hydrogen-bond geometry (Å, º)

Cg1 is the centroid of the C1–C6 ring.

**Table:**

*D*—H···*A*	*D*—H	H···*A*	*D*···*A*	*D*—H···*A*
C3—H3···N1^i^	0.95	2.56	3.383 (5)	146
C18—H18*A*···F1^ii^	0.99	2.47	3.415 (5)	159

Symmetry codes: (i) −*x*+1, −*y*+1, −*z*+1; (ii) −*x*+1, *y*−1/2, −*z*+3/2.

### 2-(Adamantan-1-yl)-5-(4-chlorophenyl)-1,3,4-oxadiazole (II) Crystal data

**Table:**

C_18_H_19_ClN_2_O	*F*(000) = 664
*M**_r_* = 314.80	*D*_x_ = 1.380 Mg m^−^^3^
Monoclinic, *P*2_1_/*n*	Cu *K*α radiation, λ = 1.54184 Å
*a* = 13.08241 (19) Å	Cell parameters from 11758 reflections
*b* = 6.49259 (9) Å	θ = 3.7–79.0°
*c* = 18.5129 (3) Å	µ = 2.25 mm^−^^1^
β = 105.5609 (16)°	*T* = 160 K
*V* = 1514.83 (4) Å^3^	Needle, colourless
*Z* = 4	0.33 × 0.12 × 0.08 mm

### 2-(Adamantan-1-yl)-5-(4-chlorophenyl)-1,3,4-oxadiazole (II) Data collection

**Table:**

XtaLAB Synergy, Dualflex, Pilatus 200K diffractometer	3217 independent reflections
Radiation source: micro-focus sealed X-ray tube, PhotonJet (Cu) X-ray Source	3052 reflections with *I* > 2σ(*I*)
Mirror monochromator	*R*_int_ = 0.023
Detector resolution: 5.8140 pixels mm^-1^	θ_max_ = 78.9°, θ_min_ = 3.7°
ω scans	*h* = −16→16
Absorption correction: analytical (CrysAlisPro; Rigaku OD, 2019)	*k* = −7→8
*T*_min_ = 0.642, *T*_max_ = 0.870	*l* = −23→23
14318 measured reflections	

### 2-(Adamantan-1-yl)-5-(4-chlorophenyl)-1,3,4-oxadiazole (II) Refinement

**Table:**

Refinement on *F*^2^	Primary atom site location: dual
Least-squares matrix: full	Hydrogen site location: inferred from neighbouring sites
*R*[*F*^2^ > 2σ(*F*^2^)] = 0.061	H-atom parameters constrained
*wR*(*F*^2^) = 0.167	*w* = 1/[σ^2^(*F*_o_^2^) + (0.094*P*)^2^ + 1.5575*P*] where *P* = (*F*_o_^2^ + 2*F*_c_^2^)/3
*S* = 1.08	(Δ/σ)_max_ < 0.001
3217 reflections	Δρ_max_ = 0.81 e Å^−^^3^
199 parameters	Δρ_min_ = −0.26 e Å^−^^3^
0 restraints	

### 2-(Adamantan-1-yl)-5-(4-chlorophenyl)-1,3,4-oxadiazole (II) Special details

**Table:**

Geometry. All esds (except the esd in the dihedral angle between two l.s. planes) are estimated using the full covariance matrix. The cell esds are taken into account individually in the estimation of esds in distances, angles and torsion angles; correlations between esds in cell parameters are only used when they are defined by crystal symmetry. An approximate (isotropic) treatment of cell esds is used for estimating esds involving l.s. planes.

### 2-(Adamantan-1-yl)-5-(4-chlorophenyl)-1,3,4-oxadiazole (II) Fractional atomic coordinates and isotropic or equivalent isotropic displacement parameters (Å^2^)

**Table:**

	*x*	*y*	*z*	*U*_iso_*/*U*_eq_	
C1	0.42626 (17)	0.6044 (4)	0.35491 (12)	0.0302 (5)	
C2	0.40263 (17)	0.4056 (4)	0.37203 (12)	0.0317 (5)	
H2	0.333320	0.351478	0.352048	0.038*	
C3	0.48137 (17)	0.2855 (4)	0.41883 (12)	0.0279 (5)	
H3	0.466239	0.148920	0.431206	0.034*	
C4	0.58338 (16)	0.3685 (3)	0.44753 (11)	0.0254 (4)	
C5	0.60437 (17)	0.5705 (3)	0.43076 (12)	0.0280 (5)	
H5	0.672884	0.627166	0.451644	0.034*	
C6	0.52611 (17)	0.6897 (4)	0.38380 (13)	0.0302 (5)	
H6	0.540617	0.826921	0.371688	0.036*	
C7	0.66623 (17)	0.2413 (3)	0.49555 (12)	0.0229 (4)	
C8	0.81754 (16)	0.1694 (3)	0.56872 (11)	0.0238 (4)	
C9	0.92972 (16)	0.2106 (3)	0.61176 (11)	0.0219 (4)	
C10	0.93691 (16)	0.3930 (4)	0.66595 (12)	0.0282 (5)	
H10A	0.897237	0.360738	0.703168	0.034*	
H10B	0.904931	0.517124	0.637722	0.034*	
C11	1.05425 (18)	0.4345 (4)	0.70636 (13)	0.0314 (5)	
H11	1.059032	0.553003	0.741610	0.038*	
C12	1.1016 (2)	0.2426 (4)	0.75071 (13)	0.0333 (5)	
H12A	1.062078	0.209782	0.787976	0.040*	
H12B	1.176566	0.268774	0.778004	0.040*	
C13	1.09521 (18)	0.0597 (4)	0.69691 (13)	0.0319 (5)	
H13	1.126743	−0.065180	0.726092	0.038*	
C14	0.97843 (17)	0.0175 (3)	0.65594 (12)	0.0285 (5)	
H14A	0.938650	−0.018439	0.692756	0.034*	
H14B	0.973710	−0.100189	0.621181	0.034*	
C15	1.11625 (17)	0.4853 (3)	0.64960 (14)	0.0319 (5)	
H15A	1.191412	0.512639	0.676075	0.038*	
H15B	1.086479	0.610423	0.621095	0.038*	
C16	1.10922 (16)	0.3031 (4)	0.59555 (13)	0.0284 (5)	
H16	1.149192	0.336599	0.557972	0.034*	
C17	1.15702 (17)	0.1113 (4)	0.63970 (14)	0.0330 (5)	
H17A	1.232475	0.136605	0.665944	0.040*	
H17B	1.153464	−0.006047	0.604985	0.040*	
C18	0.99249 (17)	0.2645 (3)	0.55507 (12)	0.0231 (4)	
H18A	0.987062	0.149794	0.518988	0.028*	
H18B	0.961848	0.389182	0.526636	0.028*	
Cl1	0.32838 (4)	0.75434 (10)	0.29606 (3)	0.0384 (2)	
N1	0.66468 (15)	0.0461 (3)	0.50869 (12)	0.0348 (5)	
N2	0.76544 (15)	−0.0012 (3)	0.55744 (12)	0.0330 (4)	
O1	0.76044 (11)	0.3299 (2)	0.53130 (8)	0.0254 (3)	

### 2-(Adamantan-1-yl)-5-(4-chlorophenyl)-1,3,4-oxadiazole (II) Atomic displacement parameters (Å^2^)

**Table:**

	*U*^11^	*U*^22^	*U*^33^	*U*^12^	*U*^13^	*U*^23^
C1	0.0236 (10)	0.0423 (13)	0.0245 (10)	0.0028 (9)	0.0060 (8)	0.0015 (9)
C2	0.0215 (9)	0.0424 (13)	0.0291 (10)	−0.0040 (9)	0.0036 (8)	−0.0017 (9)
C3	0.0218 (10)	0.0354 (11)	0.0263 (10)	−0.0056 (8)	0.0060 (8)	−0.0019 (8)
C4	0.0218 (9)	0.0291 (11)	0.0248 (9)	−0.0027 (8)	0.0054 (7)	−0.0014 (8)
C5	0.0232 (9)	0.0290 (11)	0.0307 (10)	−0.0028 (8)	0.0050 (8)	−0.0010 (8)
C6	0.0256 (10)	0.0341 (11)	0.0310 (11)	−0.0006 (9)	0.0077 (8)	0.0012 (9)
C7	0.0186 (9)	0.0250 (10)	0.0244 (10)	−0.0048 (7)	0.0045 (8)	−0.0021 (7)
C8	0.0236 (9)	0.0217 (10)	0.0263 (9)	−0.0011 (8)	0.0069 (8)	−0.0003 (8)
C9	0.0200 (9)	0.0223 (9)	0.0233 (9)	−0.0016 (7)	0.0055 (7)	−0.0007 (7)
C10	0.0248 (10)	0.0286 (11)	0.0303 (10)	0.0010 (8)	0.0056 (8)	−0.0073 (8)
C11	0.0292 (11)	0.0290 (11)	0.0311 (11)	−0.0009 (9)	−0.0001 (8)	−0.0078 (9)
C12	0.0294 (11)	0.0431 (14)	0.0233 (10)	−0.0022 (9)	−0.0001 (9)	0.0021 (8)
C13	0.0289 (11)	0.0258 (11)	0.0349 (11)	0.0003 (9)	−0.0018 (9)	0.0083 (9)
C14	0.0285 (10)	0.0229 (10)	0.0314 (10)	−0.0029 (8)	0.0034 (8)	0.0052 (8)
C15	0.0238 (10)	0.0234 (10)	0.0432 (12)	−0.0052 (8)	−0.0004 (9)	0.0036 (9)
C16	0.0205 (10)	0.0332 (11)	0.0322 (11)	−0.0003 (8)	0.0081 (8)	0.0056 (9)
C17	0.0251 (10)	0.0296 (11)	0.0413 (12)	0.0057 (9)	0.0036 (9)	0.0000 (9)
C18	0.0213 (9)	0.0254 (10)	0.0225 (9)	−0.0001 (7)	0.0059 (8)	0.0018 (7)
Cl1	0.0242 (3)	0.0543 (4)	0.0342 (3)	0.0096 (2)	0.0034 (2)	0.0102 (2)
N1	0.0268 (9)	0.0279 (10)	0.0437 (11)	−0.0052 (7)	−0.0007 (8)	0.0024 (8)
N2	0.0258 (9)	0.0255 (9)	0.0426 (11)	−0.0055 (7)	0.0000 (8)	0.0036 (8)
O1	0.0203 (7)	0.0236 (7)	0.0300 (7)	−0.0035 (6)	0.0030 (6)	0.0005 (6)

### 2-(Adamantan-1-yl)-5-(4-chlorophenyl)-1,3,4-oxadiazole (II) Geometric parameters (Å, º)

**Table:**

C1—C2	1.384 (4)	C11—H11	1.0000
C1—C6	1.387 (3)	C11—C12	1.529 (3)
C1—Cl1	1.740 (2)	C11—C15	1.526 (3)
C2—H2	0.9500	C12—H12A	0.9900
C2—C3	1.393 (3)	C12—H12B	0.9900
C3—H3	0.9500	C12—C13	1.538 (3)
C3—C4	1.405 (3)	C13—H13	1.0000
C4—C5	1.391 (3)	C13—C14	1.537 (3)
C4—C7	1.460 (3)	C13—C17	1.532 (3)
C5—H5	0.9500	C14—H14A	0.9900
C5—C6	1.388 (3)	C14—H14B	0.9900
C6—H6	0.9500	C15—H15A	0.9900
C7—N1	1.292 (3)	C15—H15B	0.9900
C7—O1	1.360 (2)	C15—C16	1.536 (3)
C8—C9	1.494 (3)	C16—H16	1.0000
C8—N2	1.288 (3)	C16—C17	1.528 (3)
C8—O1	1.359 (3)	C16—C18	1.531 (3)
C9—C10	1.538 (3)	C17—H17A	0.9900
C9—C14	1.539 (3)	C17—H17B	0.9900
C9—C18	1.536 (3)	C18—H18A	0.9900
C10—H10A	0.9900	C18—H18B	0.9900
C10—H10B	0.9900	N1—N2	1.417 (3)
C10—C11	1.540 (3)		
			
C2—C1—C6	122.0 (2)	C11—C12—C13	109.73 (18)
C2—C1—Cl1	119.56 (17)	H12A—C12—H12B	108.2
C6—C1—Cl1	118.48 (19)	C13—C12—H12A	109.7
C1—C2—H2	120.3	C13—C12—H12B	109.7
C1—C2—C3	119.4 (2)	C12—C13—H13	109.5
C3—C2—H2	120.3	C14—C13—C12	109.36 (19)
C2—C3—H3	120.3	C14—C13—H13	109.5
C2—C3—C4	119.3 (2)	C17—C13—C12	109.38 (19)
C4—C3—H3	120.3	C17—C13—H13	109.5
C3—C4—C7	119.2 (2)	C17—C13—C14	109.69 (18)
C5—C4—C3	120.1 (2)	C9—C14—H14A	109.8
C5—C4—C7	120.68 (19)	C9—C14—H14B	109.8
C4—C5—H5	119.7	C13—C14—C9	109.39 (17)
C6—C5—C4	120.6 (2)	C13—C14—H14A	109.8
C6—C5—H5	119.7	C13—C14—H14B	109.8
C1—C6—C5	118.6 (2)	H14A—C14—H14B	108.2
C1—C6—H6	120.7	C11—C15—H15A	109.8
C5—C6—H6	120.7	C11—C15—H15B	109.8
N1—C7—C4	128.6 (2)	C11—C15—C16	109.40 (18)
N1—C7—O1	112.39 (19)	H15A—C15—H15B	108.2
O1—C7—C4	119.03 (17)	C16—C15—H15A	109.8
N2—C8—C9	129.94 (19)	C16—C15—H15B	109.8
N2—C8—O1	112.47 (18)	C15—C16—H16	109.5
O1—C8—C9	117.48 (17)	C17—C16—C15	109.55 (18)
C8—C9—C10	111.42 (17)	C17—C16—H16	109.5
C8—C9—C14	110.16 (17)	C17—C16—C18	109.88 (18)
C8—C9—C18	107.76 (17)	C18—C16—C15	108.81 (17)
C10—C9—C14	109.67 (17)	C18—C16—H16	109.5
C18—C9—C10	108.65 (17)	C13—C17—H17A	109.8
C18—C9—C14	109.12 (17)	C13—C17—H17B	109.8
C9—C10—H10A	109.8	C16—C17—C13	109.39 (18)
C9—C10—H10B	109.8	C16—C17—H17A	109.8
C9—C10—C11	109.27 (17)	C16—C17—H17B	109.8
H10A—C10—H10B	108.3	H17A—C17—H17B	108.2
C11—C10—H10A	109.8	C9—C18—H18A	109.6
C11—C10—H10B	109.8	C9—C18—H18B	109.6
C10—C11—H11	109.3	C16—C18—C9	110.41 (17)
C12—C11—C10	109.02 (19)	C16—C18—H18A	109.6
C12—C11—H11	109.3	C16—C18—H18B	109.6
C15—C11—C10	110.36 (18)	H18A—C18—H18B	108.1
C15—C11—H11	109.3	C7—N1—N2	105.90 (18)
C15—C11—C12	109.41 (19)	C8—N2—N1	106.14 (18)
C11—C12—H12A	109.7	C8—O1—C7	103.10 (16)
C11—C12—H12B	109.7		

### 2-(Adamantan-1-yl)-5-(4-chlorophenyl)-1,3,4-oxadiazole (II) Hydrogen-bond geometry (Å, º)

Cg1 is the centroid of the C1–C6 ring.

**Table:**

*D*—H···*A*	*D*—H	H···*A*	*D*···*A*	*D*—H···*A*
C3—H3···N1^i^	0.95	2.61	3.386 (3)	139
C12—H12*A*···*Cg*1^ii^	0.99	2.73	3.680 (3)	160

Symmetry codes: (i) −*x*+1, −*y*, −*z*+1; (ii) *x*+1/2, −*y*+1/2, *z*+1/2.
